# Barattiere: An Italian Local Variety of *Cucumis melo* L. with Quality Traits between Melon and Cucumber

**DOI:** 10.3390/plants9050578

**Published:** 2020-05-01

**Authors:** Massimiliano Renna, Massimiliano D’Imperio, Maria Gonnella, Angelo Parente, Pietro Santamaria, Francesco Serio

**Affiliations:** 1 Institute of Sciences of Food Production (ISPA), CNR, via Amendola 122/O, 70126 Bari, Italy; massimiliano.renna@ispa.cnr.it (M.R.); maria.gonnella@ispa.cnr.it (M.G.); angelo.parente@ispa.cnr.it (A.P.); 2 Department of Agricultural and Environmental Science, University of Bari Aldo Moro, via Amendola 165/A, 70126 Bari, Italy; pietro.santamaria@uniba.it

**Keywords:** agrobiodiversity, nutritional traits, principal component analysis, unripe melon, valorisation

## Abstract

Barattiere, belonging to the *Cucumis melo* L. species, is a local variety of Puglia (Southern Italy), which is consumed as a vegetable at the immature stage, like cucumber. In this study, three Barattiere populations (‘Monopoli’, ‘Carovigno’ and ‘Fasano’) were evaluated for the main quality traits. All genotypes showed a very light green-yellow colour of flesh, without any difference regarding chlorophyll and carotenoid contents. Carovigno’s Barattiere showed the highest values of dry weight (6.8 g 100 g^−1^ fresh weight - FW), sugars (45 g kg^−1^ FW), and sweetness index (7.3), while Monopoli’s Barattiere showed the lowest total phenols content (21 mg kg^−1^ FW). Fasano’s Barattiere showed the highest content of Zn and Cu (2.3 and 0.3 mg kg^−1^ FW, respectively), while ‘Monopoli’ showed the highest Ba content (0.3 mg kg^−1^ FW) and the lowest Mg content (94 mg kg^−1^ FW). No differences between populations were found concerning the content of Ca, K, Na, B, Mn, and Fe. In conclusion, the quality profile of Barattiere makes this local genotype interesting for its traits, and also suggests its consumption by people with specific dietary requirements.

## 1. Introduction

**Figure 1:**
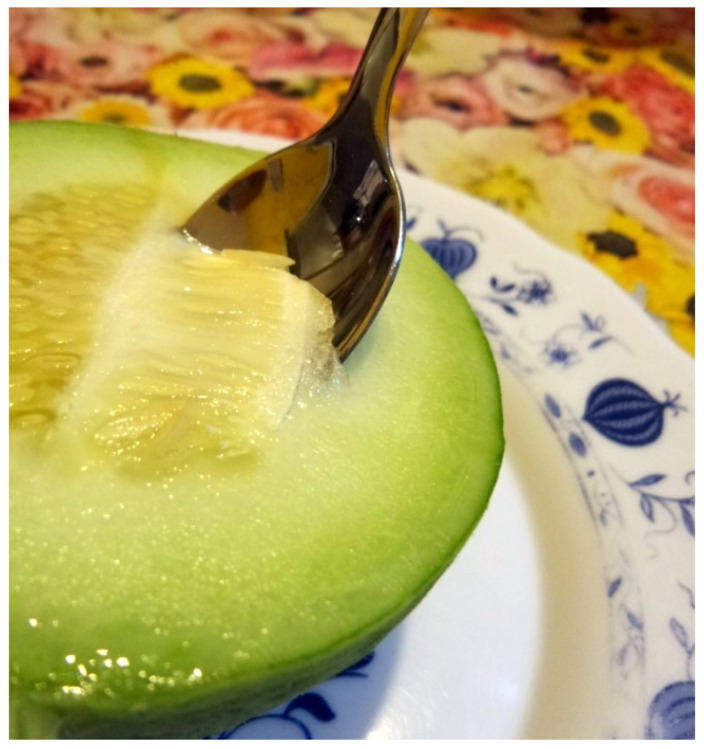
Placenta of Barattiere fruits consumed as “green caviar”.

The term Barattiere is used to indicate a Puglia (Southern Italy) local variety of *Cucumis melo* L., which is consumed unripe and is appreciated as an alternative to cucumber (*Cucumis sativus* L.), due to its better quality profile and the absence of cucurbitacin [1]. This Italian local variety has been recently introduced to a Dutch supermarket chain under the name “Cumelo” [2], a combination of the words cucumber and melon, because the product looks very much like a melon, but it greatly resembles a cucumber. Barattiere can be considered as refreshing and digestible vegetable as well as high in potassium and low in reducing sugar and sodium contents [3]. Harvesting is typically carried out when fruits have crunchy and consistent flesh texture, the seeds are still barely visible, and the placenta cavity is absent [4]. At this stage, the whole placenta can also be consumed, thus some local people have named this part of the fruit as “green caviar” (Figure 1), underlining the delicacy of the product.

To the best of our knowledge, the cultivation of Barattiere, not included in the ISTAT (Italian Institute of Statistic) survey, occurs for only few hectares (about 100 ha) in Puglia, with similar surfaces in Sicily and also little surfaces in other region of Southern (Basilicata and Campania) and, recently, Central Italy (Lazio and Tuscany). Average production for Barattiere is 30 t ha^−1^ in open field cultivation, that increases to 80-100 t ha^−1^ in greenhouse cultivation with vertical grown plants. Nowadays, it is possible to observe a highly fragmented cultivation of Barattiere, characterized by small areas in small enterprises conducted by old farmers and their families, but supported by higher technical inputs, which provide high value products for the local market and for the national and international ones as well. Therefore, the recently strong interest coming from the Dutch market for this local variety of vegetable is not unexpected [2]. Given the emerging interest for this local variety as a regional origin food product, it should also be considered to have it recognized by specific marks such as the protected designation of origin (PDO) and the protected geographical indication (PGI).

Barattiere belongs to the wide vegetable biodiversity heritage of Puglia. This region, like the entire geographical area of Southern Italy and, probably, of the Mediterranean basin, has a rich collection of vegetable genotypes, coming from an impressive selection work made by small local farmers and seed savers in the past centuries [5,6,7]. The widespread consumption of these vegetables in Puglia derives from traditional dietary habits and climate conditions that indirectly influence the diet. In fact, the hot temperature in summer and the mild climatic conditions favourable to vegetable production enhance the availability and consumption of fruits and vegetables as a relevant part of the Mediterranean diet [8].

Barattiere corresponds to the definition given in Padulosi et al. [9] for the underutilized crops which generally comprise both underutilized and neglected crops, as “genotypes known and grown since long time, saved by guardian farmers who possess a wide and heterogeneous variety of genetic biodiversity”. In the specific case of Barattiere, even if the seeds of some landraces are produced by small seed companies, there is a high risk of losing the enormous biodiversity, saved and kept by old farmers, who preserve a rich source of genes useful to improve several aspects of the crop, such as organoleptic features or the adaptability to dry climate or the tolerance to pathogens. In this context, an effective safeguard and valorization work of this underutilized crop requires its characterization, aiming to define its peculiar traits [5,10,11]. Unfortunately, although some studies on Barattiere are available [1,12,13,14,15,16,17], a quality evaluation on cultivated populations is still lacking.

Starting from these remarks, the aim of the present study was to assess the main quality and nutritional traits of three populations of Barattiere to capture the existing variability inside the typical cultivation area of this crop. The more general goal was to increase the knowledge of this local variety and promote its diffusion on the market.

## 2. Results and Discussion

### 2.1. Colour Traits

**Figure 2:**
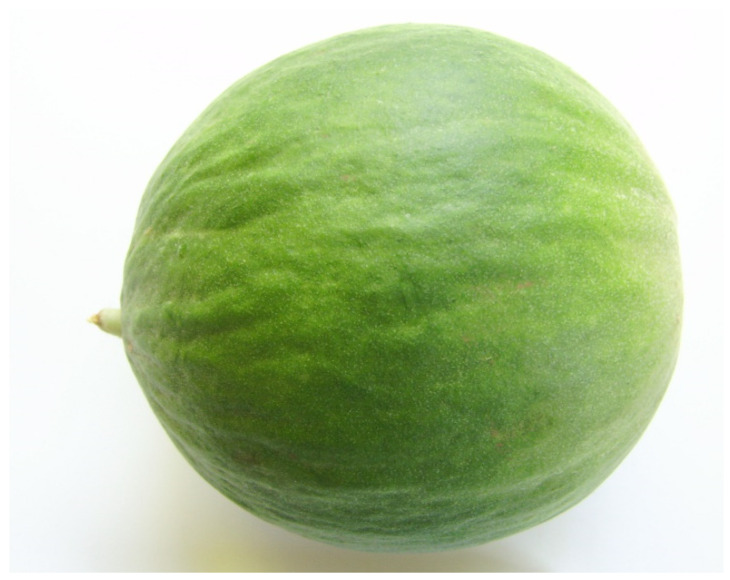
A typical Barattiere fruit ready for the market: it is interesting to note the skin colour greenish and slightly yellow.

Barattiere exocarp (skin) showed average L and h° values of 51.0 and 120.3, respectively, without differences between the populations (Table 1). At the same time, a* value was 7% lower in ‘Monopoli’ than ‘Carovigno’ and ‘Fasano’, while b* and C values were, respectively, 19% and 9% higher in ‘Monopoli’ than in the other populations (Table 1). The surface colour of food can be considered one of the most important quality parameters evaluated by consumers, since it represents the first sensation which can influence the product acceptance [18]. Considering that a* negative values indicate greenish colours, while b* positive values indicate yellowish ones, our results suggest that the Barattiere fruit skin can be described between greenish and slightly yellow (Figure 2). In particular, ‘Monopoli’ fruits can be perceived as both more “yellow” and “green” than Fasano and Carovigno (Table 1). Furthermore, consumers could perceive ‘Monopoli’ fruits by their higher colour intensity. In this context, it is interesting to note that a higher C value translates into a higher quantitative attribute of colourfulness [18].

For the mesocarp (flesh), L, a* and h° values were, on average, 75.8, 9.2 and 113.0, respectively, without differences between the populations (Table 2). ‘Monopoli’ showed b* and C values both 14% higher than ‘Carovigno’, without differences in comparison with ‘Fasano’ (Table 2).

L value can be considered as a measurement of luminosity, and it is interesting to note the much higher values of L in the flesh than in the skin of Barattiere. Therefore, our results show that the colour of Barattiere flesh can be described as a very light green-yellow, with minimal differences between the populations. It is well known that during the development of melons there is a decrease of chlorophyll content, and a simultaneous increase of carotenoids until full fruits maturity [19]. Therefore, the very light green-yellow colour of Barattiere flesh is due to the peculiarity of this vegetable, which is harvested and consumed as “unripe” melon, before the full physiological ripening. Nevertheless, it should be considered that, apart from changes in chlorophyll and carotenoids during melons development and ripening, the genotype also affects colour traits of fruit flesh. In fact, wild melon fruits show very light green flesh, while the flesh of cultivated ones can be green (due to a recessive allele *gf*), white (due to a recessive allele *wf*) or orange (presence of β-carotene, due to a dominant allele *gf*+ and/or *wf*+) [20]. Although there is a lack of information in the literature with regard to CIELab colour traits of this local variety, Elia and Santamaria [4] described the colour of Barattiere flesh as “light green” also in fully mature fruits. Therefore, our study suggests that very light green-yellow colour of this local variety can be considered a diversification trait as a result of selection by farmers during centuries. In this context, it’s interesting to highlight that a local variety may be adapted to both environmental and cultivation conditions, such as growing practices commonly used by farmers of the area where the local variety has been selected [21].

### 2.2. Dry Weight and Chemical Characteristics

‘Carovigno’ genotype showed a dry weight content 15% higher than ‘Fasano’ and ‘Monopoli’ (Table 3). Also, for glucose and fructose, ‘Carovigno’ showed the highest contents, which were, respectively, 25% and 22% higher than ‘Fasano’ and ‘Monopoli’ (Table 3). As a consequence, also for sweetness index, ‘Carovigno’ showed the highest value, which resulted 23% higher than ‘Fasano’ and ‘Monopoli’ (Table 3).

In a study that aimed to evaluate some traits of Barattiere grown in greenhouse by using a soilless system, Buttaro et al. [1] reported an average fruit dry weight of 4.95 g 100 g^−1^ FW. Since in our study all three populations were cultivated in open field, it is possible that cultivation conditions favoured a dry matter accumulation which results higher than that reported in literature [1]. Probably, the higher dry weight of ‘Carovigno’ was related to a higher amount of soluble solids such as the sugars (Table 3). This is in agreement with the strong positive correlation between dry weight and glucose content (Pearson correlation coefficient - PCC = 0.832; *p* ≤ 0.05) as well as between dry weight and fructose content (PCC = 0.866; *p* ≤ 0.05) that we obtained.

Since Barattiere looks very much like a melon, but it greatly resembles a cucumber [2], it might be interesting to compare sugar content between this local variety and cucumber and melon. Based on the average data reported by the National Nutrient Database of the United States Department of Agriculture, cucumber contains 6.3 g kg^−1^ FW of glucose and 7.5 g kg^−1^ FW of fructose [22], while melon (cantaloupe type) contains 15.4 g kg^−1^ FW of glucose, 18.7 g kg^−1^ FW of fructose and 43.5 g kg^−1^ FW of sucrose [23]. In our study on Barattiere, sucrose was not detected. From a nutritional point of view, the sugar content of Barattiere appears intermediate between cucumber and cantalupe melon, with 100 g of this local variety supplying 3.9 g of sugars (Table 3), while the same serving size of cucumber and cantalupe melon supply 1.4 and 7.8 g of sugars, respectively. Apart the nutritional aspects it could be considered that the sweetness index of Barattiere (on average 6.4) also appears intermediate between cucumber (2.3) and cantaloupe melon (11.7).

As regards total phenol content (TPC), no differences were found between ‘Carovigno’ and ‘Fasano’, which showed an average amount about three-fold higher than ‘Monopoli’ (Table 3). Phenols derive from the secondary plants metabolism and represent an important group of antioxidant compounds for human body. Since there is a lack of information in the literature on the TPC in Barattiere fruits, it may be interesting to compare the content of these antioxidant compounds among this local variety and cucumber and melon. Sotiroudis et al. [24] reported a phenol content of 138 mg kg^−1^ in cucumber flesh, while other authors [25,26] report a content ranging from 110 to 748 mg kg^−1^ in melon flesh. In all three populations of Barattiere, the TPC (Table 3) appears lower than cucumber and melon, suggesting that inside *Cucumis* spp. there is a great influence of the genotype for this quality trait.

On average, chlorophyll a and b, total chlorophyll and total carotenoids were 6.98, 6.66, 13.61, and 1.67 μg g^−1^ FW, respectively, without differences between populations (Table 4).

The lack of differences of chlorophyll content between the populations is in agreement with the lack of differences of L values (Table 1 and Table 2). Effectively, L values may be the result of different chlorophyll concentration, since vegetable colour is the result of different pigment content. In this context, Lancaster et al. [27] found a relationship between log (chlorophyll concentration) and L value, with a logarithmic relationship between increasing chlorophyll and darkness of vegetables. From a quality point of view, the content of total carotenoids of Barattiere appears slightly higher than cucumber (0.7 μg g^−1^ FW) [18] and very lower than cantaloupe melon (over 20 μg g^−1^ FW) [23]. Carotenoids are a class of yellow-orange pigments that play essential functions in plants (e.g., modulation of the photosynthetic apparatus) as well as an important role for human health. According to Clayberg [28] both wf+_/gf+_ and wf+_/gfgf genotypes produce melon fruits having orange colour flesh (due to the presence of carotenoids), the genotype wfwf/gf+_ produce fruits with white flesh, while the wfwf/gfgf genotype fruits with green flesh. The low content of carotenoids in Barattiere fruits reflect its very light green-yellow colour (see previous paragraph), probably for the absence of the dominant alleles *wf*+, which instead characterizes the orange types of *C. melo* L. [28]. This is in agreement with the absence of differences of carotenoids content between populations, suggesting that this quality parameter is not affected by different growing conditions.

As regards elements content in Barattiere fruits, ‘Fasano’ showed an amount of Mg 57% higher than ‘Monopoli’ without differences in comparison with ‘Carovigno’ (Table 5). ‘Monopoli’ showed the highest content of Ba, which were 160% and 73% higher than ‘Carovigno’ and ‘Fasano’, respectively (Table 5). ‘Fasano’ showed a content of Zn 196% and 45% higher than ‘Monopoli’ and ‘Carovigno’, respectively (Table 5). Also, for Cu, ‘Fasano’ showed the highest content, which resulted 74% higher than ‘Carovigno’ and ‘Monopoli’ (Table 5). On average, the contents of Ca, K, Na, B, Mn, and Fe were 198.1, 1749, 205.4, 1.09, 0.52, and 1.51 mg kg^−1^ FW, respectively, without differences between populations (Table 5).

**Figure 3:**
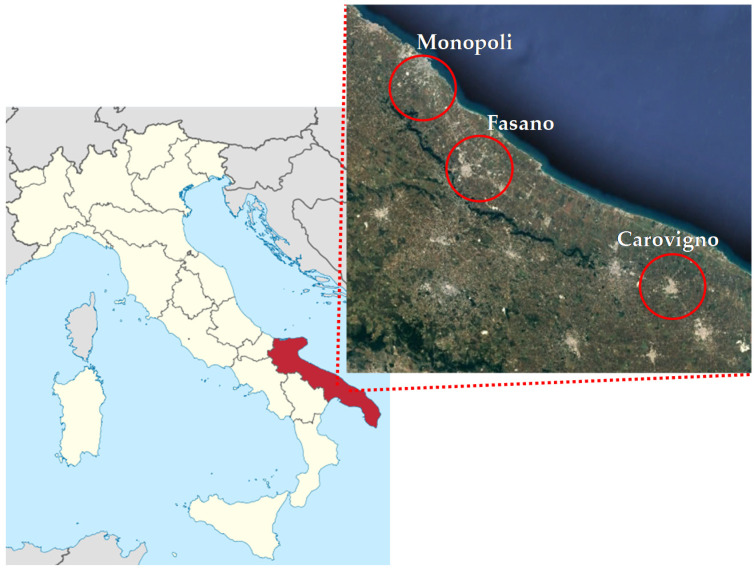
Map of Italy with Puglia region (highlighted in red) and a focus of the three sites (within the red circle) where Barattiere populations were cultivated and collected.

It should be considered that irrigation water, extracted from underground wells, was typically brackish (EC 2.8–3.0 dS m^−1^) due to the proximity of the fields to the coast. Specifically, the irrigation water used for ‘Monopoli’ came from wells closer to the coast than those of ‘Fasano’ and ‘Carovigno’ genotypes. For this reason, the irrigation water used for ‘Monopoli’ showed a higher sodium content and a lower content of calcium and magnesium than water used for ‘Fasano’ and ‘Carovigno’ genotypes (data not shown). Therefore, our results suggest that only for some elements both genotypes provenience and growing conditions can influence their content in Barattiere fruits. We found a strong positive correlation between TPC and Zn content (PCC = 0.734; p ≤ 0.05). ‘Monopoli’ genotype showed the lowest TPC (Table 3) and the lowest Zn content in fruits (Table 5). In concordance, in a study aimed to evaluate the chemical profile of some different local varieties of tomato, Renna et al. [11] found the highest TPC in fruits which showed a higher Zn amount than other ones. In another study that aimed to evaluate the effect of Zn biofortication in *Brassica oleracea* L., Barrameda-Medina et al. [29] found that an increased Zn concentration in plant tissue increased the phenols content. These results suggest a correlation between TPC content in Monopoli fruits (Table 3) and Zn content than other Barattiere populations (Table 5). Apart the correlation between TPC and Zn, we found that TPC showed a strong positive correlation with Mg (PCC = 0.745; p ≤ 0.05) and K (PCC = 0.644; p ≤ 0.05) as well as a strong negative correlation with Ba (PCC = −0.676; p ≤ 0.05). From a nutritional point of view, for all populations, fruits showed a Ca content higher than those reported by the National Nutrient Database of the United States Department of Agriculture for cucumber (140 mg kg^−1^ FW) and cantalupe melon (90 mg kg^−1^ FW) [22,23]. As regards K, the content appears intermediate between cucumber and melon, with 100 g of this local variety supplying 175 mg of K (Table 5), while the same serving size of cucumber and cantalupe melon supply 136 and 267 mg, respectively [22,23]. K is an essential nutrient for the human body, since it is well known that its optimal intake is associated with lower blood pressure. At the same time, for people affected by chronic kidney disease the K intake requires to be restricted in order to avoid phenomena of hyperkalemia [30]. Therefore, although Serio et al. [3] described Barattiere as a vegetable with high K content, the consumption of this local variety could be preferred to the common melon when the restriction of high-K foods is recommended. Contrary to that reported for K, Barattiere is described as vegetable with low Na content [3]. Nevertheless, the different populations described in this study showed an amount of Na content higher than those reported by the National Nutrient Database of the United States Department of Agriculture for cucumber (20 mg kg^−1^ FW) and cantalupe melon (160 mg kg^−1^ FW) [22,23]. Santamaria et al. [31] reported a higher Na content in tomato fruits by using a nutrient solution with a high electrical conductivity (EC). In our study, all Barattiere populations were grown in fields near the coast (Figure 3), by irrigation with brackish water extracted from underground wells. Therefore, it is possible that the EC values of irrigation water may have influenced the Na content of Barattiere fruits. Anyway, as reported by the European Food Safety Authority, the daily required intake for Na corresponds to 1500 mg [32]. Therefore, the Na content found in the three Barattiere populations could be considered negligible from a nutritional point of view. On the other hand, the possibility to increase or decrease the mineral elements in Barattiere fruits could be considered by applying agronomic biofortification strategies [33,34] as a valorization tool for this local variety.

### 2.3. Principal Component Analysis

**Figure 4:**
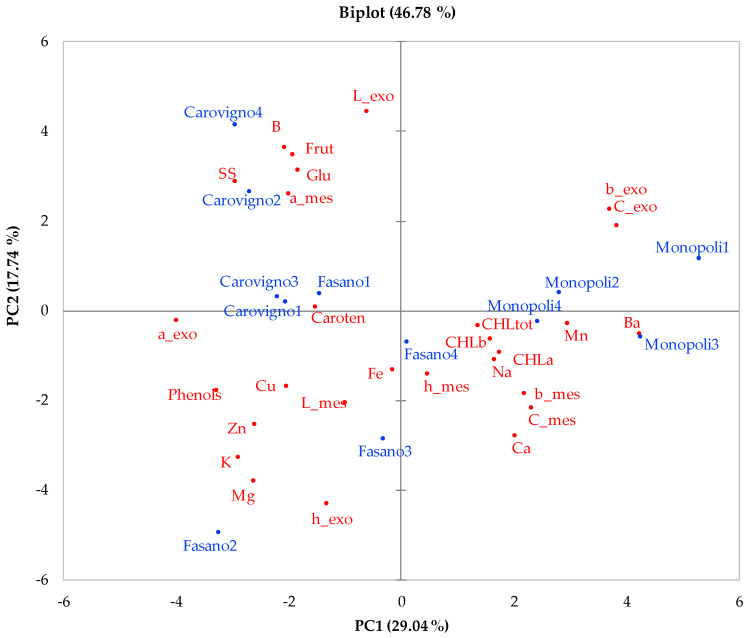
PCA biplot (Principal Component 1 - PC1 vs. Principal Component 2 -PC2) describing the variation of the physical and chemical parameters measured to characterize the three studied Barattiere populations. For each population, numbers 1, 2, 3 and 4 indicate different replicates. L, a, b, C and h denote *L*, a*, b*, C, and h°, respectively, as colour parameters; exo, exocarp; mes, mesocarp; Caroten, total carotenoids; total phenols content, TPC; Frut, fructose; Glu, glucose; CHL, chlorophyll; tot, total; B, boron; Ba, barium; Ca, calcium; Cu, copper; Zn, zinc; Mg, magnesium; Mn, manganese; Na, sodium; K, potassium; Fe, iron.

The first five principal components (PCs) explained 80% of the total variance, with principal component 1 (PC1) and principal component 2 (PC2) accounting for 29% and 18%, respectively. The PCA biplot (Figure 4) shows the relationships between the parameters considered in this study. Parameters located close to each other had a strong co-variance, and parameters far from the origin contributed more to the PCs than ones close to it. PC1 was correlated with colour parameters (negatively by exocarp a* and positively by exocarp b* and exocarp C) as well as with TPC, dry weight, K, Mg, and Zn (negatively) and with Ba and Mn (positively) (Figure 4). PC2 was correlated with exocarp L, B, fructose, glucose and dry weight (positively) and with exocarp h°, Mg, K and Ca (negatively). The PCA biplot also revealed a separation between ‘Carovigno’ (located on the negative side of PC1) and ‘Monopoli’ (located on the positive side of PC1) (Figure 4). The first population is characterized by high dry weight and sugars, while ‘Monopoli’ was distinguished by high C values and Ba content as well as low TPC, confirming the results of ANOVA.

## 3. Materials and Methods

### 3.1. Location and Cropping Details

**Figure 5:**
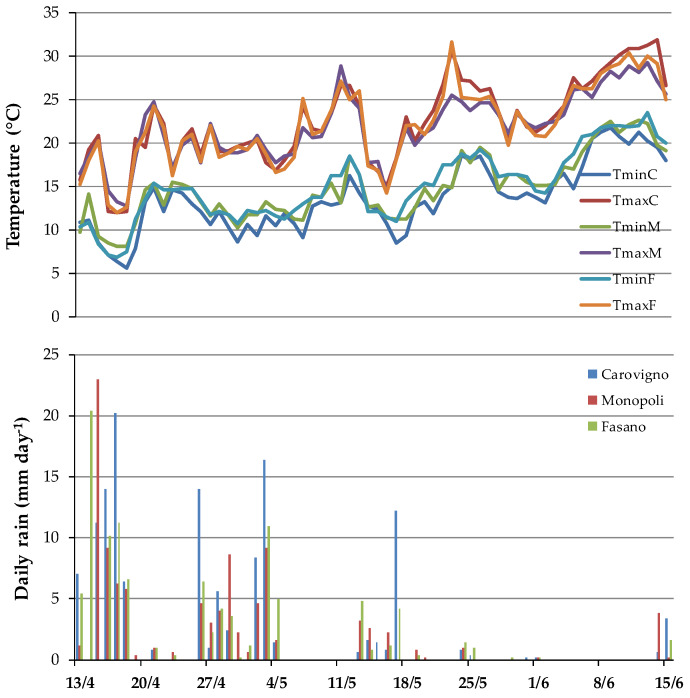
Temperatures and daily rain during the growing cycle. T, temperature; min, minimum; max, maximum; C, ‘Carovigno’; M, ‘ Monopoli’, F, ‘Fasano’.

Three Barattiere populations were open field cultivated on local farms according to the best management practices of the integrated farming system. Each genotype was cultivated inside the area where it has been selected: ‘Monopoli’ (40°55′5.8” N 17°19′40.8” E), ‘Fasano’ (40°51′27.8” N 17°24′16.3” E), and ‘Carovigno’ (40°44′28.8” N 17°42′46.9” E) (Figure 3), since each population is characterized by a specific adaptation to both environmental and local cultivation conditions. Therefore, each population has a local name that corresponds to the name of the cultivation site. Seeds for crop propagation were self-produced and stored on-farm by each farmer. Transplanting (distance of 1 m between the rows and 0.3 m on the row) was carried out on 15 April 2014 for all sites, and growing techniques were in line with the agricultural practices of local farmers specialized in growing the Barattiere. In detail, referred to as average description, fertilization was made giving N–P–K as 100/120–60/80–60/80 kg ha^−1^, respectively, for the most part in pre-transplanting and the residuals as N fertilizers supplied in fertigation during the cycle. Plants were cultivated on silty-sandy soil, typical of the Puglia coastal areas, under the Mediterranean climate, with rain almost absent and maximum temperatures sometimes approaching 30–35 °C on the hottest days during the summer season. In the period between transplant and the harvest of fruits for our samples, only supplemental irrigation was needed and applied as rescue drip-irrigation, for a total water amount of 50 mm applied according to crop needs (see rain and temperatures patterns–Figure 5). Irrigation water, extracted from underground wells placed near the coast (500–2000 m away), had an electrical conductivity (EC) of about 2.8–3.0 dS m^−1^.

Harvesting took place on 15 June for all sites. Sampling was carried out by harvesting the first fruit on the main stem when it reached the typical form and size (equatorial diameter of about 10 cm), according to the BBCH-scale for cucurbits (code 701) [35]. For each population a total of 30 fruits (10 fruits x 3 replications) were harvested (one per plant). After the harvest, the samples were refrigerated and immediately transported to the laboratory to be processed and analyzed as described in the following sections.

### 3.2. Physical Analysis

Barattiere fruits were cut longitudinally and the colour of their external and internal surfaces was measured by using the CIELAB scale (L, a*, b*) with a portable tristimulus colour-meter (Minolta Chroma Meter CR-400; Minolta Camera Co. Ltd., Osaka, Japan), according to the procedure described by Renna et al. [36]. The instrument runs with the colour-space coordinates designed as: L, “lightness” (ranging from black = 0 to white = 100); a*, “red/green chromaticity”; b*, “yellow/blue chromaticity”. Through trigonometric functions, other colour indices were calculated: (i) colour saturation, C = [(a*)^2^ + (b*)^2^]^1/2^; (ii) hue angle, h° = tan^−1^ (b*/a*). Before the measurements, the colorimeter was calibrated with a standard reference with L, a*, and b* values of 97.01, 0.10 and 1.88, respectively.

For the measurement of dry weight (DW), fresh samples (half part of each fruit) were maintained in a forced draft oven at 105 °C until constant weight was reached.

### 3.3. Samples Preparation for Chemical Analysis

For each replication Barattiere fruit samples (the residual half part of each fruit) were freeze-dried by a LABCONCO FreeZone^®^ Freeze Dry System, model 7754030, (Kansas City, MI, USA) equipped with a LABCONCO FreeZone^®^ Stoppering Tray Dryer, model 7,948,030 (Kansas City, MI, USA). The freeze-dried samples were ground at 500 μm by using a Retsch laboratory mill (Torre Boldone, BG, Italy) to obtain a homogeneous powder.

### 3.4. Glucose and Fructose Assay, and Sweetness Index

Glucose and fructose content were determined by ionic chromatography (Dionex model DX500; Dionex Corp., Sunnyvale, CA, USA) using a pulsed amperometric detector (PAD) according to protocols used by Renna et al. [11]. Peak separation was performed using a Dionex CarboPac PA1 and isocratic elution with 50 mmol L^−1^ NaOH. Results were expressed as mg g^−1^ FW.

The sweetness index (SI) was calculated based on the content and sweetness properties of individual carbohydrates by multiplying the sweetness coefficient of each sugar (glucose = 1.00, fructose = 2.30 and sucrose = 1.35) by the concentration (g 100 g^−1^ FW) of that sugar in fruits [37]. The following formula was used:

SI = (g glucose 100 g^−1^ FW) ∗ 1.00 + (g fructose 100 g^−1^ FW) ∗ 2.30 + (g sucrose 100 g^−1^ FW) ∗ 1.35 (1)

### 3.5. Total Phenols Content

The TPC was determined by using the Folin Ciocalteu method reported by D’Imperio et al. [38]. Briefly, approximately 0.2 g of lyophilized sample were mixed with solvent mixture (MeOH:H_2_O:CH_3_COOH 79:20:1% v/v/v). The vials were then placed in a sonicator bath at ambient temperature for 30 min, followed by 1 h in magnetic stirred. The sample was centrifuged at 10,000× *g* for 10 min at 4 °C and the supernatant was transferred into a volumetric tube. The residue was resuspended in fresh solvent mixture, gently mixed manually and sonicated for 30 min followed stirring (1 h) and centrifugation (10,000× *g* for 10 min at 4 °C). The two supernatants were combined, and appropriate aliquots of extracts were filtered (0.45 μm). TPC was determined using gallic acid (R^2^ = 0.9998) as a calibration standard by using a Perkin-Elmer Lambda 25 spectrophotometer (Boston, MA, USA).

### 3.6. Chlorophylls and Total Carotenoid Content

Chlorophylls and carotenoid were determined spectrophotometrically using the extraction procedure reported by Renna et al. [39]. Fresh samples were homogenized in 80% acetone, and the absorbance of the extract was measured at 662, 645, and 470 nm with a UV-1800 spectrophotometer (Perkin-Elmer Lambda 25, Boston, MA, USA).

### 3.7. Elemental Analysis

Macro and microelements (Ca, K, Mg, Na, B, Mn, Ba, Zn, Fe, and Cu) concentrations were determined according to D’Imperio et al. [40]. Briefly, a representative amount (0.25 g) of freeze-dried powder samples was digested in a closed-vessel microwave digestion system (MARS 6, CEM Corporation, Matthews, NC, USA) with 65% HNO_3_ (10 mL). The microwave digestion protocol was applied in two steps: 15 min up to 200 °C and 10 min at 200 °C stable (power set at 900–1050 W; 800 psi) by using HNO_3_ without sample as blanks. After mineralization, the samples, diluted with ultrapure H_2_O (Milli-Q Millipore 18 M W/cm), were filtered using a 0.45 μm filter. The mineralized samples were analysed with the Agilent 5100 Vertical Dual View ICP-OES (Santa Clara, CA, USA) to measure Ca, K, Mg, and Na in radial mode and the minor elements (B, Mn, Ba, Zn, Fe, and Cu) in axial mode.

### 3.8. Statistical Analysis

A one-way analysis of variance (ANOVA) was performed using the General Linear Model (GLM) procedure (SAS software, Version 9.1). The separation of means was obtained by the least significant difference (LSD) test. For a visual analysis of data, principal component analysis (PCA) (XLStat, Addinsoft, Paris, France) was performed on mean centred and standardized (unit variance scaled) data prior to analysis. The data matrix submitted to PCA was made up of 12 observations (three genotypes per four replications) and 28 variables. PCCs (PROC CORR, SAS software, Cary, NC, USA) were used to quantify relationships between dependent variables.

## 4. Conclusions

Barattiere is an Italian local variety of *Cucumis melo* L. (unripe melon) used as a vegetable which shows a skin between greenish and slightly yellow and a flesh that is very light green-yellow, with very small differences among populations present locally. The sugar content in this local variety can be considered intermediate within those of cucumber and cantaloupe melon, while TPC appears lower than both cucumber and cantaloupe melon, although this quality trait may vary among populations. Independently of the population, Barattiere fruits showed a Ca content higher than those reported for cucumber and cantaloupe melon, while K content appears intermediate between cucumber and cantaloupe melon. In conclusion, the quality profile of Barattiere makes this local variety interesting for its nutritional traits, suggesting its consumption as an alternative to other vegetables such as cucumber. This example of agro-biodiversity can be considered part of a vegetable heritage worth saving. Moreover, its valorisation may represent a small achievement in pursuit of the diversification of the current food market. Future research activities may be directed toward its comprehensive characterization by using molecular tools.

## Figures and Tables

**Table 1:** Colour parameters of Barattiere exocarp.

Population	L	a^*^	b^*^	h^°^	C
Carovigno	53.1	−17.34 a	30.0 b	120.1	34.6 b
Fasano	49.7	−17.69 a	29.6 b	120.8	34.5 b
Monopoli	50.3	−18.78 b	32.5 a	120.0	37.5 a
*Significance*	ns	*	*	ns	*

Significance: ns = not significant; * significant for *p* ≤ 0.05. Different letters indicate statistically significant differences at *p* = 0.05. L, lightness; a*, red/green chromaticity; b*, yellow/blue chromaticity; C, colour saturation; h°, hue angle.

**Table 2:** Colour parameters of Barattiere mesocarp.

Population	L	a^*^	b^*^	h^°^	C
Carovigno	76.4	−8.6	20.2 b	113.0	22.0 b
Fasano	75.8	−9.1	21.5 ab	112.9	23.4 ab
Monopoli	75.3	−9.8	23.0 a	113.1	25.0 a
*Significance*	ns	ns	*	ns	*

Significance: ns = not significant; * significant for *p* ≤ 0.05. Different letters indicate statistically significant differences at *p* = 0.05. L, lightness; a*, red/green chromaticity; b*, yellow/blue chromaticity; C, colour saturation; h°, hue angle.

**Table 3:** Dry weight (DW), sugars, sweetness index (SI) and total phenols content (TPC) of Barattiere fruits.

Population	DW	Glucose	Fructose	SI	TPC
	g 100 g^−1^ FW	g kg^−1^ FW			mg kg^−1^ FW
Carovigno	6.77 a	23.3 a	21.8 a	7.35 a	62.83 a
Fasano	5.94 b	18.0 b	17.3 b	5.77 b	66.80 a
Monopoli	5.89 b	19.3 b	18.5 b	6.21 b	21.01 b
*Significance*	*	*	*	**	*

Significance: * and ** significant for *p* ≤ 0.05 and *p* ≤ 0.01, respectively. Different letters indicate statistically significant differences at *p* = 0.05.

**Table 4:** Chlorophylls and total carotenoids content of Barattiere fruits.

Population	Chlorophyll a	Chlorophyll b	Total Chlorophyll	Total Carotenoids
	μg g^−1^ FW			
Carovigno	6.25	6.25	12.50	1.88
Fasano	7.02	6.58	13.60	1.56
Monopoli	7.67	7.07	14.74	1.57
*Significance*	ns	ns	ns	ns

Significance: ns = not significant.

**Table 5:** Mineral elements content of Barattiere fruits.

Population	Ca	K	Mg	Na	B	Mn	Ba	Zn	Fe	Cu
	mg kg^−1^ FW									
Carovigno	170.3	1729	113.8 ab	175.3	1.17	0.38	0.10 c	1.57 b	1.46	0.18 b
Fasano	199.3	2170	148.2 a	165.0	1.08	0.53	0.15 b	2.28 a	1.65	0.27 a
Monopoli	224.8	1347	94.0 b	276.0	1.01	0.64	0.26 a	0.77 c	1.41	0.13 b
*Significance*	ns	ns	*	ns	ns	ns	***	***	ns	*

Significance: ns = not significant; * and *** significant for *p* ≤ 0.05 and *p* ≤ 0.001, respectively. Different letters indicate statistically significant differences at *p* = 0.05.
